# Robotic-assisted percutaneous coronary intervention: current evidence and future directions

**DOI:** 10.3389/fcvm.2026.1827660

**Published:** 2026-07-01

**Authors:** Benjamin Bay, Moritz Seiffert, Fabian J. Brunner

**Affiliations:** 1 Department of Cardiology, University Heart and Vascular Center Hamburg, University Medical Center Hamburg-Eppendorf, Hamburg, Germany; 2 Center for Population Health Innovation (POINT), University Heart and Vascular Center Hamburg, University Medical Center Hamburg-Eppendorf, Hamburg, Germany; 3 German Center for Cardiovascular Research (DZHK), Partner Site North, Hamburg, Germany; 4 Department of Cardiology and Angiology, BG University Hospital Bergmannsheil, Ruhr-University Bochum, Bochum, Germany

**Keywords:** advanced coronary interventions, complex PCI, coronary arter disease, PCI—percutaneous coronary intervention, robotic interventions

## Introduction

**Figure 1:**
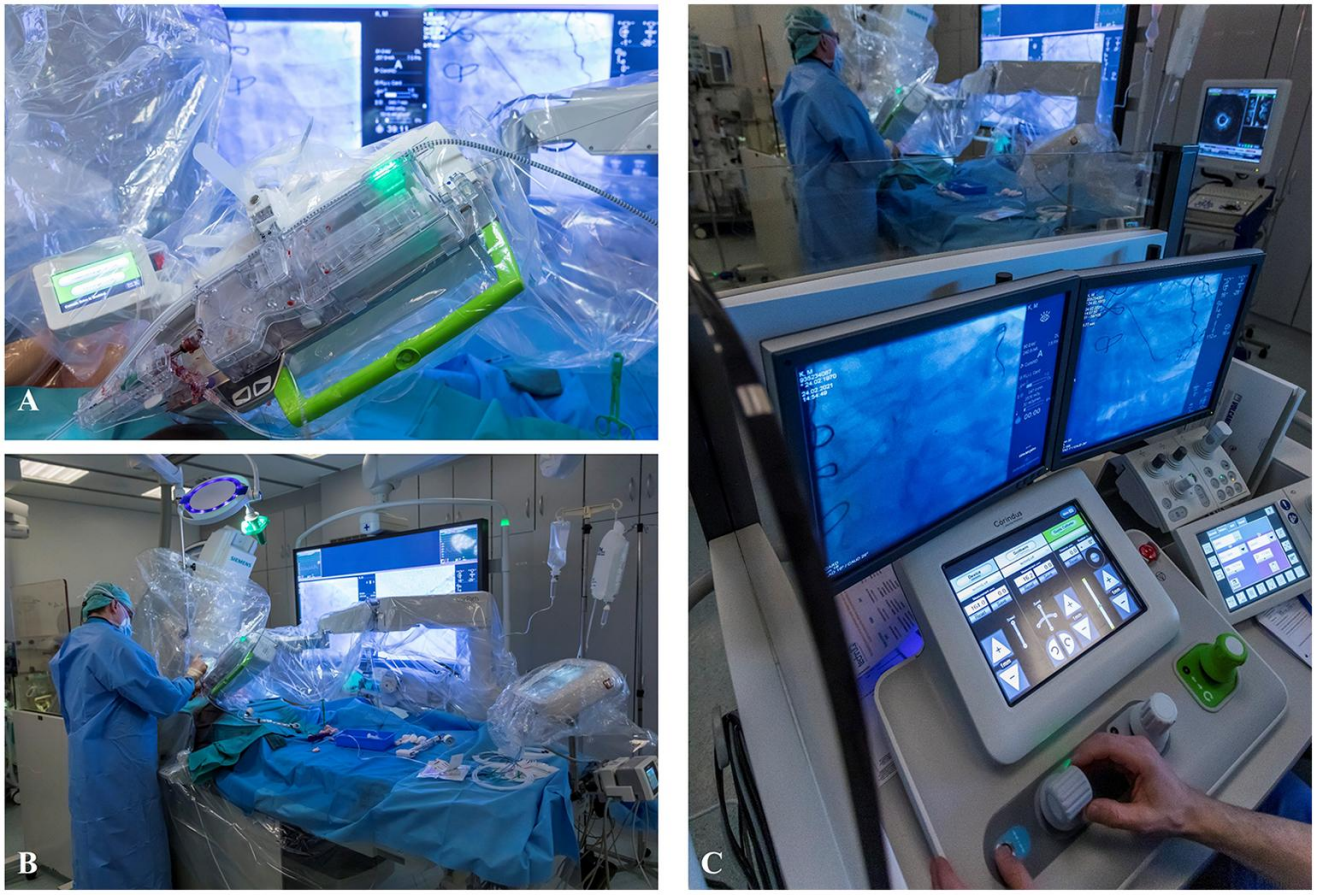
The rPCI platform and catheterization laboratory configuration. The robotic arm, loaded with a disposable cassette and connected to the arterial sheath and guiding catheter, enables robotic-assisted wire and device navigation **(A)** Wire and device exchanges are carried out by a bedside assistant **(B)**The operator's workstation provides remote control of the robotic system. Physical separation between the assistant and the c-arm is facilitated by the setup, reducing occupational radiation exposure **(C)** rPCI, robotic-assisted percutaneous coronary intervention.

Manual percutaneous coronary intervention (mPCI), i.e., conventional PCI, is a catheter-based revascularization technique in which the operator manually advances guidewires, balloons, and stents through the coronary vasculature under fluoroscopic guidance, with direct bedside control of all devices and inherent occupational radiation exposure. mPCI has become the mainstay for coronary revascularization across the spectrum of coronary artery disease (CAD) (1). In chronic coronary syndromes, PCI is, in particular, indicated for symptom relief for hemodynamically relevant stenoses, but also recommended in dedicated anatomies for prognostic indication (2). In acute coronary syndromes, including Non-ST-segment elevation myocardial infarction (NSTEMI) and STEMI, an invasive strategy using PCI is commonly implemented as primary reperfusion approach (3). Within this scope, the ISCHEMIA trial most certainly reinforces the importance of a tailored management to individual patients and encourages us to explore a more conservative approach if possible when considering the management of CCS especially in patients in whom the risk of an invasive strategy may outweigh any potential symptomatic benefit (4). Despite the undeniable benefit for the patient treated, mPCI is also associated with relevant occupational risks for the operators. Chronic exposure to ionizing radiation persists despite modern dose-reduction strategies and has been linked to an increased risk for malignancy and cataracts for interventionalists (5–7). In addition, prolonged procedures performed under heavy lead protection contribute to musculoskeletal disorders, with implications for long-term operator health and career longevity (8, 9). Also, novel shielding solutions eliminating the weight burden for the operator have not yet become widely adopted (10, 11). In contrast to mPCI, robotic-assisted PCI (rPCI) transfers device manipulation to a computer-controlled robotic arm directed from a shielded remote workstation, enabling sub-millimeter guidewire and device navigation outside the primary radiation field using a dedicated interface (Figure 1). Over the last two decades, several rPCI platforms have been developed (First-generation CorPath 200 [Corindus, USA]; Second-generation CorPath GRX [Corindus, USA]; R-One robotic system [Robocath, France]; ETcath200 [Beijing WeMed Medical Equipment, China]), building on the success of the robotic approach for surgical indications (12, 13). These rPCI systems may be a way to reduce these occupational health hazards for the interventionalists and, ideally, should deliver patient outcomes at least equivalent to those achieved with mPCI, at least in appropriately selected cases of non-complex CAD.

## rPCI—clinical applicability and basic principles

In general, rPCI is performed using dedicated robotic platforms which translate operator inputs into intracoronary device movements. While different rPCI systems have been described, the procedural approach follows a common core principle (14–17). A robotic arm is positioned at the cardiac catheterization table and equipped with a sterile, disposable cassette that interfaces with interventional hardware. After manual vascular access and engagement of the coronary ostium, the guide catheter and sheath are connected to the robotic cassette, enabling subsequent robotic control of guidewires and delivery systems. The primary operator conducts the intervention remotely from a shielded interventional cockpit or from a workstation located in the control room using a dedicated console to advance, retract, or rotate devices with high precision. A scrubbed assistant is positioned at the bedside to set up the robotic arm, load guidewires, balloons, or stents into the cassette, reposition the catheterization table under fluoroscopy and perform contrast injections as needed.

Based on these technical differences in contrast to mPCI, the robotic-assisted approach offers several potential advantages for interventionalists as well as patients. These include operator radiation reduction and improved ergonomics for the operator as well as the potential for tele-robotic PCI. The idea of a possible enhancement of procedural precision, however, needs to be evaluated more thoroughly in this context (13).

## Procedural results and technical success

After the initial description of the rPCI approach, a continuous development has been described in the literature, utilizing rPCI in different lesion complexities and clinical scenarios. Pivotal studies for the first-generation platforms include the Percutaneous Robotically Enhanced Coronary Intervention (PRECISE) and Complex Robotically Assisted Percutaneous Coronary Intervention (CORA-PCI) multicenter registries (18, 19). In the PRECISE study the first-generation CorPath 200 system was assessed in 164 patients with low-complexity coronary lesions (68.4% classified as type A or B1), demonstrating a high procedural success rate of 97.6% (18). In contrast, the CORA-PCI study included a large proportion of complex lesions (80% classified as type B2 or C), underscoring the potential use of the rPCI platform across the spectrum of coronary interventions (19). More recent, the safety and efficacy of newer-generation robotic systems, incorporating automated guidewire control, whilst also using the robotic-assisted approach in increasingly anatomically complex lesions has been demonstrated in several registry based analyses (15, 17, 20–22).

Nonetheless, whilst multiple technological improvements have expanded the ability to treat increasingly complex coronary lesions using a robotic-assisted approach, several limitations persist (13). In a contemporary multi-center cohort of patients who underwent rPCI using the latest available second generation CorPath GRX System, manual support (either manual assistance or conversion to mPCI) was needed in *n* = 46 (19.9%) of the *n* = 231 treated coronary lesions. Manual support was more likely in lesions involving the left anterior descending artery, aorto-ostial lesions, chronic total occlusions, true bifurcations, and severe calcification (23). This rate is similar to that reported for earlier CorPath systems, in which *n* = 20 of *n* = 108 rPCI procedures (18.5%) required some form of manual involvement (24). Similarly, for other contemporary rPCI platforms such as the R-One and ETCath systems comparable or even higher rates of full manual conversion were reported (17, 22).

Aside from procedural success, multiple studies have shown a substantial reduction in operator radiation exposure, measured with personal dosimeters, using both the original Corindus platform and the newer R-One system (17, 25) The comparable efficacy, safety and improved radiation dose for the operator is however associated with prolonged procedural times [mPCI: 27 min (Interquartile range: 21–40) vs. rPCI: 37 min (Interquartile range: 27–50); *p* < 0.0005] for the treated patients (26, 27). This may in part be attributable to the learning curve of the primary operator and is expected to improve with increasing experience in rPCI procedures. The literature suggests a threshold of approximately 50 rPCI cases, after which trained interventionalists achieve procedure times comparable to those of mPCI (26).

## Outcomes after rPCI

Available literature suggests that rPCI has comparable short-term and mid-term clinical outcomes to mPCI in selected patients, albeit without clinical superiority. With the increasing use of rPCI, first comparative analyses of both procedural aspects as well as clinical outcomes were described in 2019 and 2020 for the first-generation platforms. Here, the PRECISE study reported very high technical and clinical success with excellent 30-day safety, and the CORA-PCI study in more complex lesions found similar clinical success to mPCI despite longer procedure times (18, 19). For the latest generation CorPath GRX, multicenter registry data confirmed persistently high clinical success in real-world practice, while several contemporary cohort studies showed no significant difference vs. mPCI in 1-year mortality or MACE (20, 21, 28). Similar results are documented for the R-One platform (17). Early data for the ETcath200 data are promising but still preliminary (single-center, small sample), showing no 30-day adverse events (22). A recent meta-analysis analyzing the outcomes of the different published registry reports across the different rPCI platforms underscored these findings. Here, in a total of 10 investigated studies, MACE and all-cause mortality were comparable between mPCI and rPCI (29).

## Discussion

The concept underlying the development of rPCI platforms is highly appealing. Potential advantages are (i) reduction of radiation exposure to the operator, (ii) more ergonomic working environment with decreased musculoskeletal strain, (iii) the possibility of remote-controlled PCI support via long-range distances, and (iv) greater procedural precision, analogous to robotic-assisted surgical interventions.

The most immediate benefit of rPCI is a reduction in radiation exposure for the primary operator, via the increased spatial distance from the radiation source during fluoroscopy (17, 25) However, the clinical relevance of this advantage should be interpreted in the context of the entire catheterization team and the patient. In routine practice, a second operator typically remains scrubbed at the table to perform tasks that cannot be completed robotically (e.g., vascular access, guide catheter engagement and stabilization, device exchange support, management of acute instability, etc.) (14–17). This is underscored by recent data which display a high rate of manual support, and thus the need for an assistant who is scrubbed in, even for the newer generation platforms (23). Therefore, the second operator and further staff still require conventional personal protective equipment, with its well-known physical burden. Alternatives such as non-person related radiation protections systems available (e.g., suspended or cabin-based radiation protection solutions such as Rampart and Zero-Gravity) are not yet universally implemented and currently do not represent a valid alternative with regard to the reduction in musculoskeletal strain as well as radiation exposure (10, 11) Additionally, for the treated patient, longer fluoroscopy times (rPCI: 20.4 min vs. mPCI: 14.4 min), albeit with comparable dose-area products between groups, were recently documented in a pooled analysis from 4 centers using the CorPath GRX System (20). Previously, a reduced dose-area product has been reported for rPCI vs. mPCI procedures (27). Hence, any radiation benefit for assisting operators, further staff as well as for the patient is less certain.

The concept of remote PCI, i.e., so called “tele-stenting” is another frequently cited potential advantage of rPCI, and pilot studies have described its feasibility in transregional and transcontinental settings (30–32). In selected scenarios, remote support by an experienced operator may indeed be attractive, especially to expand access to expertise. However, broad implementation in real-world PCI appears challenging. As described above, key procedural steps including non-crossable lesions remain manual and therefore operator-dependent. In addition, patient deterioration or hemodynamic instability and other potential complications require prompt hands-on intervention. These limitations are especially relevant for complex coronary interventions, which represent a key potential application for rPCI, but in which manual support is more often necessary (23). In this regard, the need for on-site expertise remains despite the hypothetical use of rPCI platforms. In specific clinical contexts, i.e., settings where trained support personnel is available but no interventionalist is on site, tele-stenting represents a conceptually viable application of rPCI technology.

Current technical limitations of rPCI platforms remain substantial and directly affect everyday clinical applicability. Over the past two decades, substantial advancements in robotic platforms for the treatment of stenotic CAD have led to clinical outcomes comparable to those of mPCI. However, in routine clinical practice, rPCI has not yet been widely adopted. As noted, none of the currently available and former rPCI systems allow simultaneous manipulation of multiple guidewires, balloons, or stents. Moreover, fully robotic operation of over-the-wire devices—such as atherectomy systems or microcatheters—is not possible and necessitates manual support (13). Intravascular imaging modalities (e.g., intravascular ultrasound and optical coherence tomography) can also only be partially utilized and often cumbersome in robotic workflows (33). This is particularly relevant because contemporary PCI practice is becoming more complex and increasingly imaging-guided, which has been shown to lead to improved outcomes after PCI (34). With regard to technical advances for rPCI platforms, one of the key improvements introduced with the second-generation CorPath GRX system was the TechnIQ software. This technology utilized smart automation to standardize and automate device movements used in manual PCI, aiming to facilitate guidewire navigation and lesion crossing, particularly in complex interventions. This included several motions, which were tested in the randomized Navigate GRX study, where a total of 50 lesions were treated either using the TechnIQ software vs. the control-cohort where the TechnIQ software was not used. Here, angiographic and clinical outcomes did not differ, whereas procedural time was reduced in the subgroup where smart automation was utilized (35). However, despite this potential advantage in clinical routine the loss of tactile feedback during coronary interventions is a further issue. In mPCI, wire resistance and catheter feel provide important information during lesion crossing and device manipulation. In rPCI, this haptic information is mostly absent, and operators must rely more heavily on visual cues and fluoroscopic behavior. Potentially, visual pattern recognition and procedural experience will be able to minimize the impact of the loss of tactile feedback, although further investigations will be needed in this space. Notably, the Chinese ETcath200 rPCI platform, as a first-in-class system, incorporates guidewire force sensing to deliver real-time tactile feedback to the operator (16, 22).

Another proposed strength of rPCI is improved procedural precision akin to the utilization of the robotic approach in surgical disciplines. Robotic systems allow controlled and reproducible advancement/retraction of guidewires and devices, which may theoretically improve lesion crossing and stent positioning accuracy. However, evidence demonstrating superior clinical outcomes due to increased precision in rPCI vs. mPCI is lacking. Previously, a lower rate of longitudinal geographical miss during stent placement as well as a reduction in the number of used stents was noted using the rPCI approach (36, 37). In the future, integration of advanced intraprocedural imaging, preprocedural Coronary-CT, and potentially AI-assisted decision making might lead to improved outcomes. Nonetheless, the potential improved precision of stent placement during rPCI represents a technological promise rather than a proven advantage.

**Figure 2:**
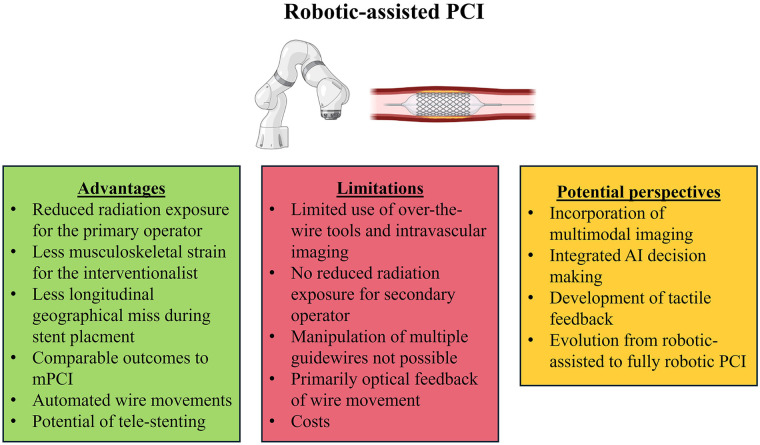
Advantages, limitations and future promise of robot-assisted PCI platforms. mPCI, manual percutaneous coronary intervention.

Finally, future progress in rPCI will depend not only on technological innovation but also on practical implementation as well as economic considerations. Platform development and technical refinement will require routine clinical use. However, adoption is hampered by high material and infrastructure costs, and the absence of dedicated reimbursement in many healthcare systems, thus creating a structural barrier. Advantages, Limitations and potential perspectives are described in Figure 2.

The above-named limitations have led to the removal of the first- and second-generation robotic systems from Corindus, which are no longer available for clinical use. Currently, next to the available rPCI platforms from RoboCath and Beijing WeMed Medical Equipment, several novel cardiovascular robotic systems are in development or are being tested for first in man clinical use, albeit most are planned for the interventional treatment of cerebrovascular or peripheral disease rather than CAD (38, 39).

With the current technological possibilities, a widespread application of rPCI across both simple and complex coronary lesions, as well as the potential concept of tele-stenting, where PCI is performed remotely, currently seems far-fetched. The transition from robotic-assisted toward fully robotic PCI will require technological advances, while simultaneously reducing the economic burden for hospitals and healthcare providers and improving patient outcomes. However, should these advances materialize, a broad adoption of rPCI akin to the expansion of robotic techniques in the surgical field currently might be feasible.
